# Supplementary material for: Water Structures Reveal Local Hydrophobicity on the In2O3(111) Surface
Source: ACS Nano. 2022 Nov 30;16(12):21163–73. doi: 10.1021/acsnano.2c09115 (PMC9798908; doi:10.1021/acsnano.2c09115)
Supplement: Supplementary file 1 — nn2c09115_si_001.pdf [file nn2c09115_si_001.pdf]

Supporting Information

Water Structures Reveal Local Hydrophobicity on the $In_2O_3(111)$ Surface

Hao Chen[1,2,3], Matthias A. Blatnik[1,4], Christian L. Ritterhoff[5], Igor Sokolović[1], Francesca Mirabella[1], Giada Franceschi[1], Michele Riva[1], Michael Schmid[1], Jan Čechal[4], Bernd Meyer[5], Ulrike Diebold[1], Margareta Wagner[1,4]

[1]Institute of Applied Physics, TU Wien,
Wiedner Hauptstraße 8-10/134, A-1040 Vienna (Austria)

[2]State Key Laboratory of Catalysis, Dalian Institute of Chemical Physics (DICP), Chinese Academy of Sciences (CAS), 457 Zhongshan Road, Dalian 116023 (China)

[3]University of Chinese Academy of Sciences, Beijing 100049 (China)

[4]Central European Institute of Technology (CEITEC), Brno University of Technology, Purkynova 123, 612 00 Brno (Czech Republic)

[5]Interdisciplinary Center for Molecular Materials (ICMM) and Computer Chemistry Center (CCC), Friedrich-Alexander-Universität Erlangen-Nürnberg (FAU), Nägelsbachstraße 25, 91052 Erlangen (Germany)

Email: wagner@iap.tuwien.ac.at
bernd.meyer@chemie.uni-erlangen.de

Supporting Information:

Additional Experimental Data

1) Temperature Programmed Desorption S-3

2) TPD: Sticking Probability Curve of Water on $In_2O_3(111)$ S-4

3) TPD: Multilayer Desorption S-5

4) XPS Peak Fitting S-6

5) STM: Low Water Coverage as Function of Temperature S-8

6) STM and AFM: The β Phase S-9

7) Simulated AFM images of water structures S-10

Additional DFT Results

8) Structures with 18 Adsorbed Water Molecules (β Phase) S-12

9) Structures with 9 Adsorbed Water Molecules (RE Phase) S-16

10) Water Adsorption in Region A S-18

11) MD Simulation for a Thick Water Film on $In_2O_3(111)$ S-21

Additional Experimental Data

1) Temperature Programmed Desorption

The calibrated molecular beam allows exposing the sample to a controlled and precise amount of water by adjusting the beam flux (1.05×10^{13} molecules $cm^{-2}{\cdot}s^{-1}$ in our experiments) and exposure time. A photograph of an In_2O_3 thin-film sample with a thick layer of water ice is shown in Figure S1.

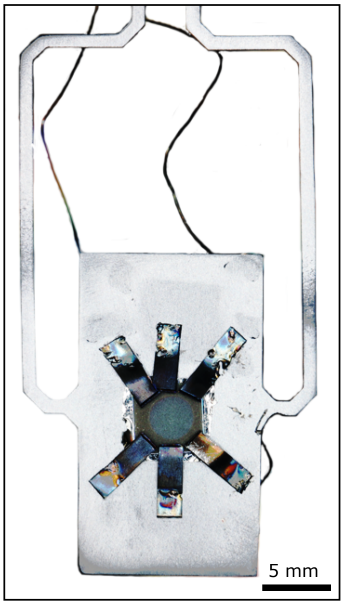

Figure S1. In_2O_3(111) thin-film sample used for TPD and XPS measurements after adsorption of a thick layer of water ice (>600 L) at ≈120 K. The size of the molecular beam is visible as a circular blue-green area with a diameter of ≈3.1 mm, centered on the sample.

2) TPD: Sticking Probability Curve of Water on $In_2O_3(111)$

Figure S2 displays a measurement of the sticking coefficient of water on $In_2O_3(111)$. In this experiment, isotopically labeled D_2O with constant flux was dosed onto the sample at normal incidence, with the mass spectrometer positioned at 45° from the surface normal, i.e., in line-of sight geometry.[35] When the sample is kept at a low temperature (132 K), the very low signal at $m = 20$, ≈300 counts/s after a total dose of 10.0 L (Langmuir, 1 L = 1×10^{-6} torr·s), implies that water sticks to the surface without any re-desorption. At a higher sample temperature, during cool-down from 470 K to 245 K, the molecules scatter from the sample directly into the mass spec. The signal rises to ≈10,000 counts, indicating a much smaller sticking coefficient.

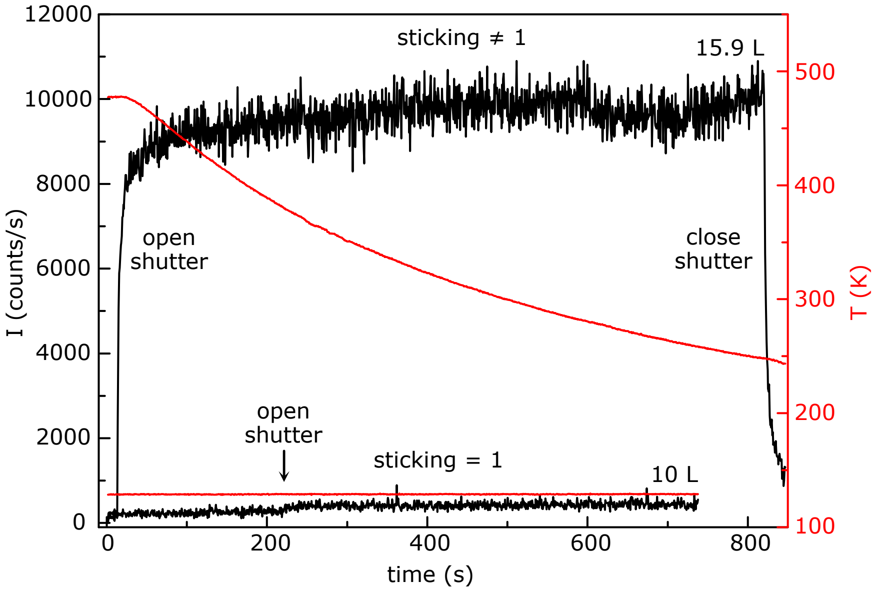

Figure S2. Sticking probability of D_2O on $In_2O_3(111)$ at different temperatures. Mass 20 signal measured during exposures to a total of 15.9 L during cooling from 470 K to 245 K (upper trace), and 10.0 L at 132 K (lower trace).

3) TPD: Multilayer Desorption

The multilayer desorption energy was evaluated from TPD curves with higher coverages than those displayed in Figure 2 of the main text. Figure S3 shows the desorption curves from $In_2O_3(111)$ after water doses of 10 and 15 L.

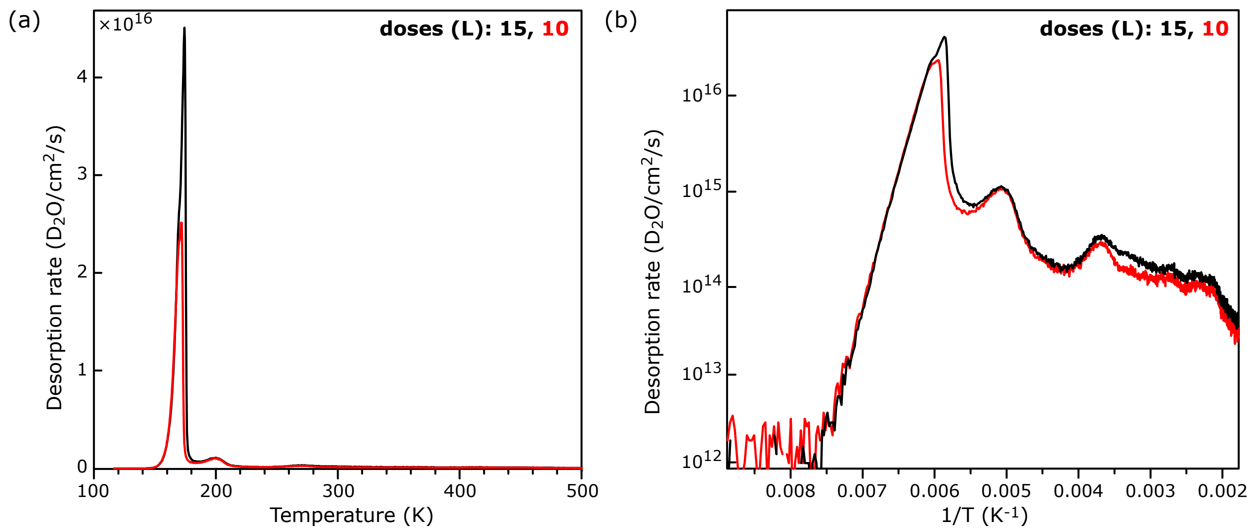

Figure S3. Multilayer desorption of D_2O from $In_2O_3(111)$, showing the leading edge of the zero-order desorption. The curves are shown in (a) linear, and (b) logarithmic scale. Heating rate: $1\,Ks^{-1}$.

4) XPS Peak Fitting

All fitting parameters of the XPS O1s core level for the fits displayed in Figure 3 of the main text are listed in Table S1. Figure S4 summarizes the integral area of the individual fitting components as a function of water molecules per surface unit cell. At 450 K, after desorbing most water species of the ζ desorption peak, a small amount of water is still present on the surface, see Figure S5. This remaining water decreases further with heating to 500 K and is associated with water at defect sites (hence the non-zero desorption rate at 500 K). The XPS spectrum obtained at 300 K shows the pristine surface prior to the water exposure.

T	TPD	$In3d_{5/2}$	FWHM	O1s	FWHM	Area	O1s OH	FWHM	Area	O1s mol-I	FWHM	Area	O1s mol-II	FWHM	Area
132	α	445.018	1.31	530.496	1.10 |f	2.9352	532 |l	1.490 |l	0.5821 |u	532.860 |l	1.490 |B	0.4972	533.98 |-	1.550 |u	1.4280
153	β	445.017	1.32	530.492	1.115 |f	3.2600	532 |l	1.498 |f	0.5821 |u	532.860 |l	1.498 |B	0.4760	533.97 |u	1.477 |-	0.7132
190	RE	445.015	1.35	530.465	1.119 |f	3.5750	532 |l	1.700 |u	0.5821 |u	532.846 |-	1.700 |B	0.4785	533.97 |u	1.700 |u	0.1108
227	γ	445.006	1.36	530.438	1.14 |u	3.7071	532.16 |l	1.750 |u	0.5890 |u	532.862 |-	1.750 |B	0.2772			
290	δ	444.955	1.36	530.382	1.16 |l	3.9460	532.17 |-	1.764 |-	0.5894 |-						
345	ε	444.915	1.34	530.353	1.199 |-	4.0937	532.12 |l	1.770 |l	0.4250 |u						
405	ζ	444.878	1.36	530.320	1.247 |-	4.2468	532 |l	1.748 |-	0.2560 |-						
450	D	444.868	1.38	530.308	1.289 |-	4.33	532 |l	1.7059 |-	0.1508 |-						
500	D	444.859	1.40	530.287	1.306 |-	4.43	532 |l	1.748 |u	0.1285 |-						
300	clean			530.367	1.216 |-	4.27									

Table S1. XPS fitting parameters of the UHV experiments. The peak shape is modelled by a Gaussian/Lorentzian product formula with a weighting of G:L = 70:30. The actual XPS curves and fits are shown in the main text. D denotes water on defect sites. Temperature T is in K, all core electron binding energies are in eV. Constraints: l (lower bound), u (upper bound), f (value fixed), B (same as for O1s OH).

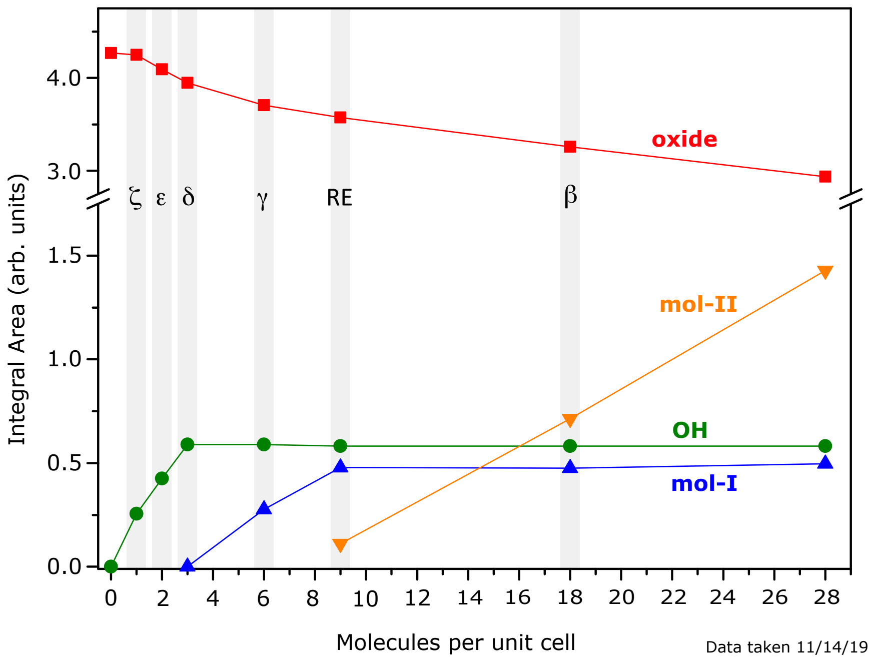

Figure S4. Peak areas of the various components listed in Table S1.

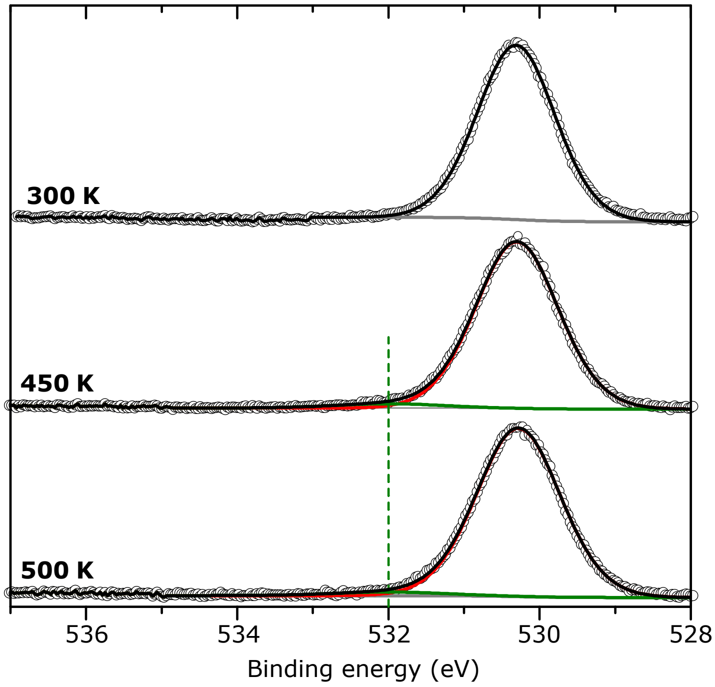

Figure S5. O1s core level after water exposure and heating the surface to 450 K and 500 K, compared to the pristine surface measured at 300 K. The fitting parameters are listed in Table S1.

5) STM: Low Water Coverage as Function of Temperature

The desorption of water from $In_2O_3(111)$ was also investigated with STM (images taken at 80 K). To this end, 1 L of water was dosed at room temperature and subsequently desorbed from the surface by heating the sample in steps of ≈20 K for 3 min each; the evolution of the water structures is shown in Figure S6. The interpretation of dissociated water structures in STM was established in previous work.[26,27] At ≈348 K, the first H_2O desorbs from the surface; almost all of the bright triangles (indicated in Figure S6 by yellow triangles) that represent three dissociated H_2O molecules per unit cell are gone. Mostly curved features (green lines), i.e., two H_2O per unit cell, are left on the surface. Desorption of the second water molecule happens around 408 K, where the curved features lose one water molecule and turn into oval features. The third water molecule is almost gone a 473 K. The temperatures confirm the assignment of the δ, ε, and ζ peaks to desorption of 3, 2, and 1 D_2O/u.c. (see Figure 2 in the main text).

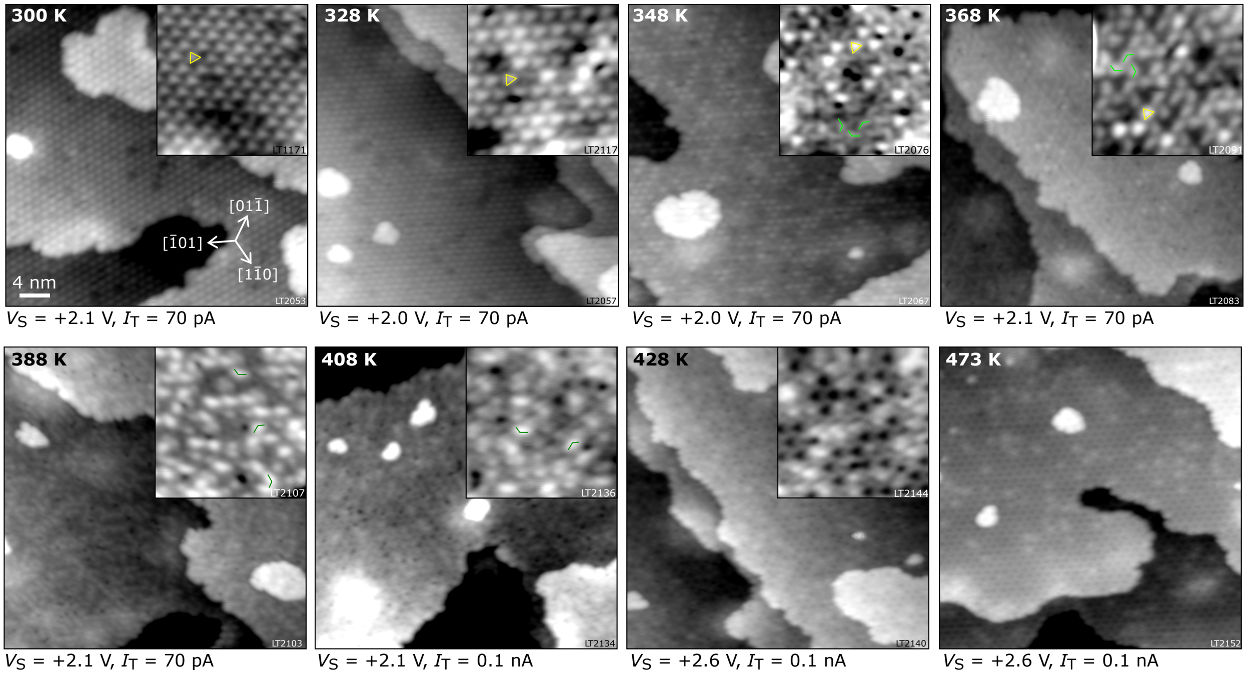

Figure S6. Water on $In_2O_3(111)$ as function of temperature, imaged with STM at 80 K.

6) STM and AFM: The β Phase

The β structure contains a total of 18 water molecules/u.c., i.e., 9 molecules in addition to the RE structure. In AFM, only the topmost water molecules are visible. They are organized in a slightly disordered, hexagonal pattern (see main text). Comparison with STM images of the same surface region shows that the protrusions are located at the corners of the unit cell (In(6c) regions, labeled B in Figure 1 of the main text). An STM image is provided in Figure S7a. The bright features correspond to the hydroxyl groups (yellow triangles in Figure S6) and are stable upon STM imaging. The molecular water is responsible for the inhomogeneous contrast and the horizontal lines where the tip interacted with them. The protrusions seen in the AFM images (obtained with zero bias voltage; see main text) change positions when the area is scanned repeatedly by STM, but they do not change in number.

AFM was employed on a sample where many of the terminating water molecules were missing, this allows to probe the layer underlying the bright protrusions. We found a variety of small clusters located predominantly in region B, see Figure S7b. Hence, we propose terminating water clusters in region B of the unit cell for the water monolayer coverage (18 molecules/u.c.) on $In_2O_3(111)$.

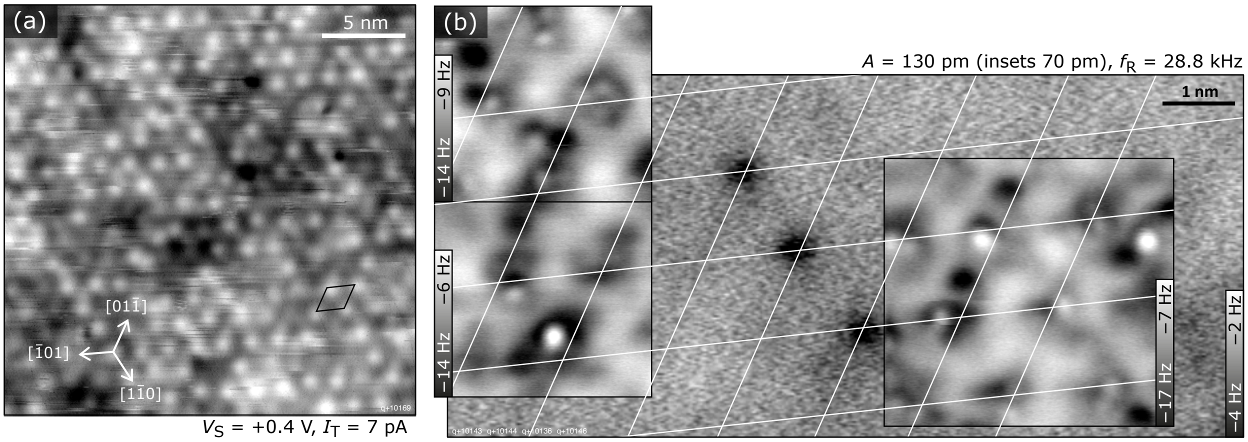

Figure S7. The β structure. (a) STM image. The protrusions correlate with the bright triangles of the hydroxylated surface. (b) Constant-height AFM images. The corners of the grid are located in the regions B of the surface unit cell. The image in the background shows three protrusions, which are the same features as in the left inset in Figure 6a of the main text, but imaged at greater distance from the surface. This results in dark (attractive) contrast of the protruding H_2O molecules. The insets in (b) were taken with the tip closer to the sample by >300 pm, in areas where the highest protrusions are missing.

7) Simulated AFM images of water structures

The optimized DFT structures for 3, 6, 9 and 18 water molecules per $In_2O_3(111)$ unit cell shown in Figures 4–6 of the main text were used to simulate constant-height AFM images. The simulated images were created using the Probe Particle Model[36,37] without the inclusion of the Hartree contribution. That is, the AFM contrasts in Figure S8 were constructed by using the empirical Lennard-Jones potentials alone. The contrast is created by simulating the interaction of an O-terminated AFM tip (CO tip) with the surface species using an oscillation amplitude of $A = 100\,\text{pm}$, the electronic charge of the tip equal to $q_{\text{tip}} = -0.05\,\text{e}^-$, and the lateral and vertical spring constant of the tip equal to $k_x = k_y = 0.5\,\text{N/m}$ and $k_z = 20\,\text{N/m}$, respectively.

The interpretation of the simulated AFM contrast in Figure S8 is similar to the interpretation of the contrast in experimental constant-height AFM images: the attractive interaction (negative frequency shift) between the tip and the sample is depicted as dark, while the bright contrast (positive frequency shift) corresponds to either repulsive tip–sample interaction or the weak long-range background interactions.

The level of detail in the simulated images in Figure S8 depends on the geometric arrangement of the water species, since the constant-height AFM images are most sensitive to the top-most protruding species. In that light, the majority of adsorbate species can be imaged in structures with the less water molecules (3 per unit cell), as their distribution is relatively planar. With the addition of water molecules, the adsorbates rearrange in vertical direction to accommodate for the presence of additional molecules, resulting in structures with some water molecules protruding further from the surface compared to the OH groups. These species are dominating the contrast, which is well exemplified in the case of 18 water molecules per unit cell, where only a single water molecule per unit cell can be imaged clearly. At the intermediate water coverages (6 and 9 water molecules per unit cell) the majority of the molecules can still be imaged. For 6 water molecules/u.c., contrast inversion upon the reduction of the tip–sample distance appears first on the three higher-lying water molecules in region C, while for 9 water molecules/u.c. the pairs formed by an O_wH (now in an on-top position) and a water molecule at the rims of the unit cells experience repulsion first.

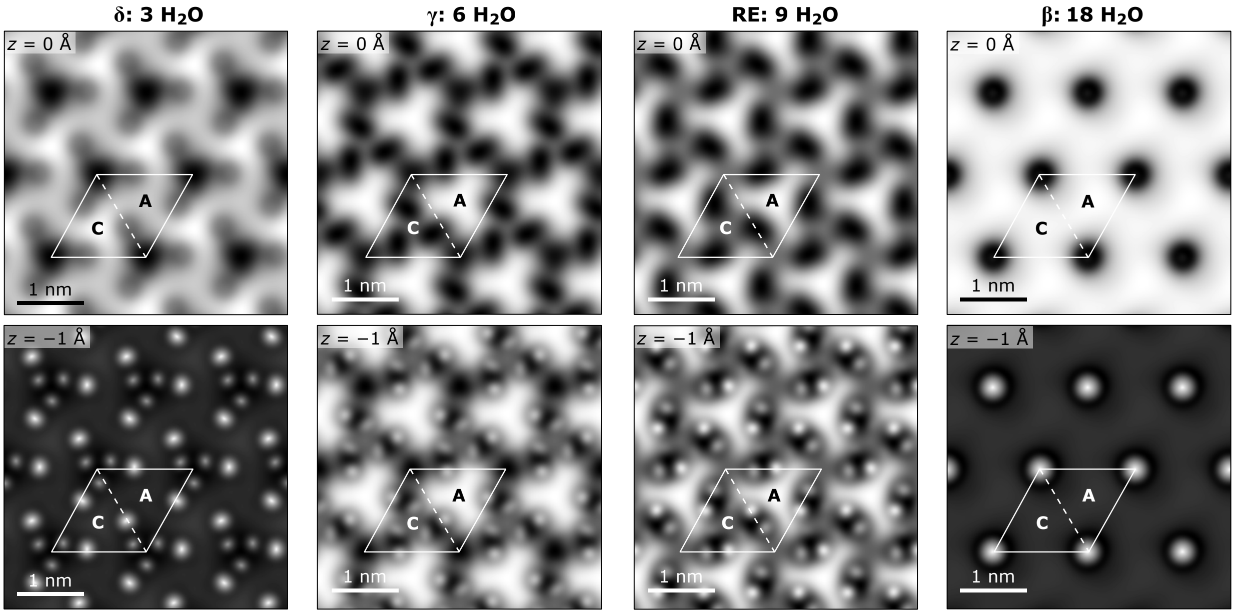

Figure S8. Simulated constant-height AFM images of different water structures and coverages. For each coverage, simulations were performed at larger (top row) and 1 Å smaller (bottom row) tip–sample distances. The larger tip–sample distance is normalized to zero separately for each structure, as different water coverages result in different vertical rearrangement of molecules. A contrast inversion from attraction to repulsion is common for most adsorbates upon the reduction of the tip–sample distance.

Additional DFT Results

8) Structures with 18 Adsorbed Water Molecules (β Phase)

In our simulated annealing structure search by *ab initio* molecular dynamics simulations we started from the hydroxylated surface structure with 6 OH groups from 3 dissociated water, to which we added 15 more intact water molecules at random positions above the surface. This structure was equilibrated for 10 ps at 360 K using massive Nosé–Hoover thermostats. In the production run of 80 ps at the same temperature, a pronounced diffusion of the water molecules was observed. At intervals of 10 ps, a configuration was taken from the trajectory and quenched to 100 K using a linear temperature ramp, followed by a final geometry optimization. Three different quenching rates were applied with 15, 45 and 90 ps for the 250 K temperature drop. Altogether, we obtained an ensemble of 24 relaxed configurations of 18 water molecules on $In_2O_3(111)$ by this simulated annealing procedure.

The lowest-energy structure of this search is shown in Figure 6 of the manuscript and in Figure S9a. The average binding energy of the 9 water molecules of the second water layer above area B and C is 0.68 eV, as calculated by removing the 9 molecules from the surface and relaxing the remaining structure. Some selected low-energy configurations of the structure search are displayed in Figure S9b-f. In some structures, one out of the 9 molecules in the second layer (Figure S9b,d) or the first layer (Figure S9f) moved to an In-a site. In all these cases, the water molecule at In-a stabilizes the O_wH group of a fourth dissociated water molecule. This is the only configuration that can support a single water molecule at one of the three In-a sites. There was never a second water molecule at an In-a site in any of our 24 structures from the simulated annealing search. Somewhat more frequently (in about 1/3 of the structures of our ensemble), one water molecule of the second layer formed an H bond to an O(α), see Figure S9c,e,f. Such H bonds were also found with a probability of about 30 % in the molecular dynamics simulation of the liquid water/$In_2O_3(111)$ interface, see Section 11. A water molecule with an OH group sticking out of the second layer, which may cause the protrusion seen in AFM, was found in almost all configurations. However, this water molecule is often not sitting in the center of the cap above site B, but is also found off-center or even at the rim of the second layer, see Figure S9b-c. In one case, there were even two water molecules with an outward-oriented OH group (Figure S9e), and in another case there was none at all (Figure S9f). However, the last two structures are already 0.1 eV higher in energy than the global optimum.

As a further confirmation that indeed a structure with water-free In-a sites and a small water cluster above B and C is the global energy minimum, the lowest-energy structures of the simulated annealing search were manually modified by shifting water molecules to and from In-a or O(α) sites and by adjusting the H-bond network by flipping the orientation of

some other water molecules. Only a few attempts resulted in different configurations with a similar or slightly lower energy than the best structure from the simulated annealing search, see Figure S10. In all cases, energy is gained either by moving a water molecule out of area A or by optimizing the H-bond network in the first layer (see also next section). We never succeeded to lower the overall energy by shifting water molecules to area A. In the end, the best structure with a water molecule adsorbed at an In-a (Figure S10a) is 0.18 eV higher in energy than the lowest-energy structure with a water-free area A (Figure S10f).

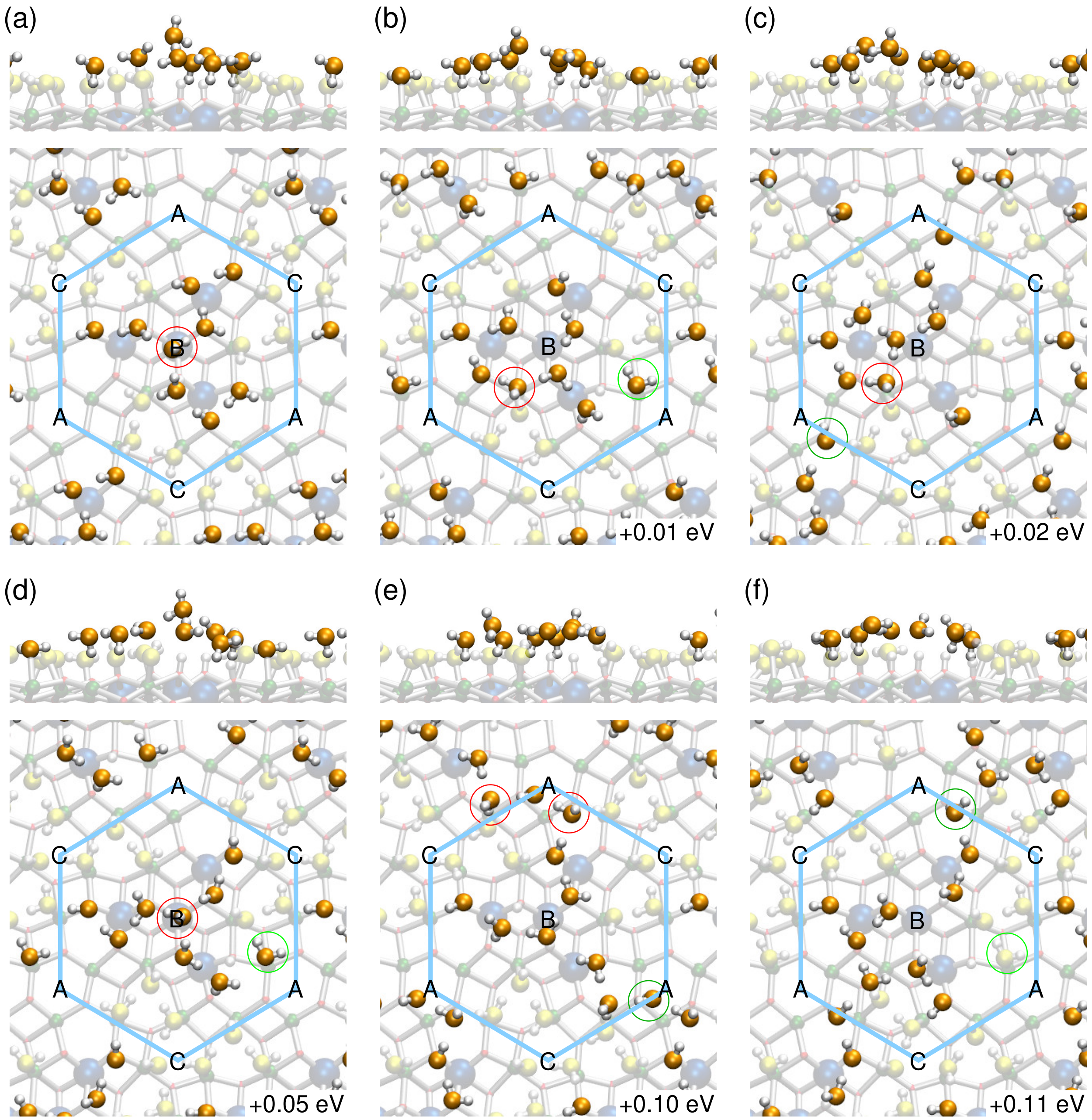

Figure S9. Selected low-energy structures of 18 water molecules on $In_2O_3(111)$ from the simulated annealing search. The energy difference per unit cell with respect to the global optimum, shown in (a), is given in the insets. The O atom of water molecules in the first and second layer are depicted in yellow and orange, respectively. Water molecules adsorbed on In-a sites or forming a H bond to an O(α) are marked by light and dark green circles, respectively, and water molecules with an OH group sticking out of the second layer are highlighted by a red circle. The fourth dissociated water molecule is at the same position as shown in Figure S11d-i. The Wigner-Seitz cell centered at site B is shown by light blue lines.

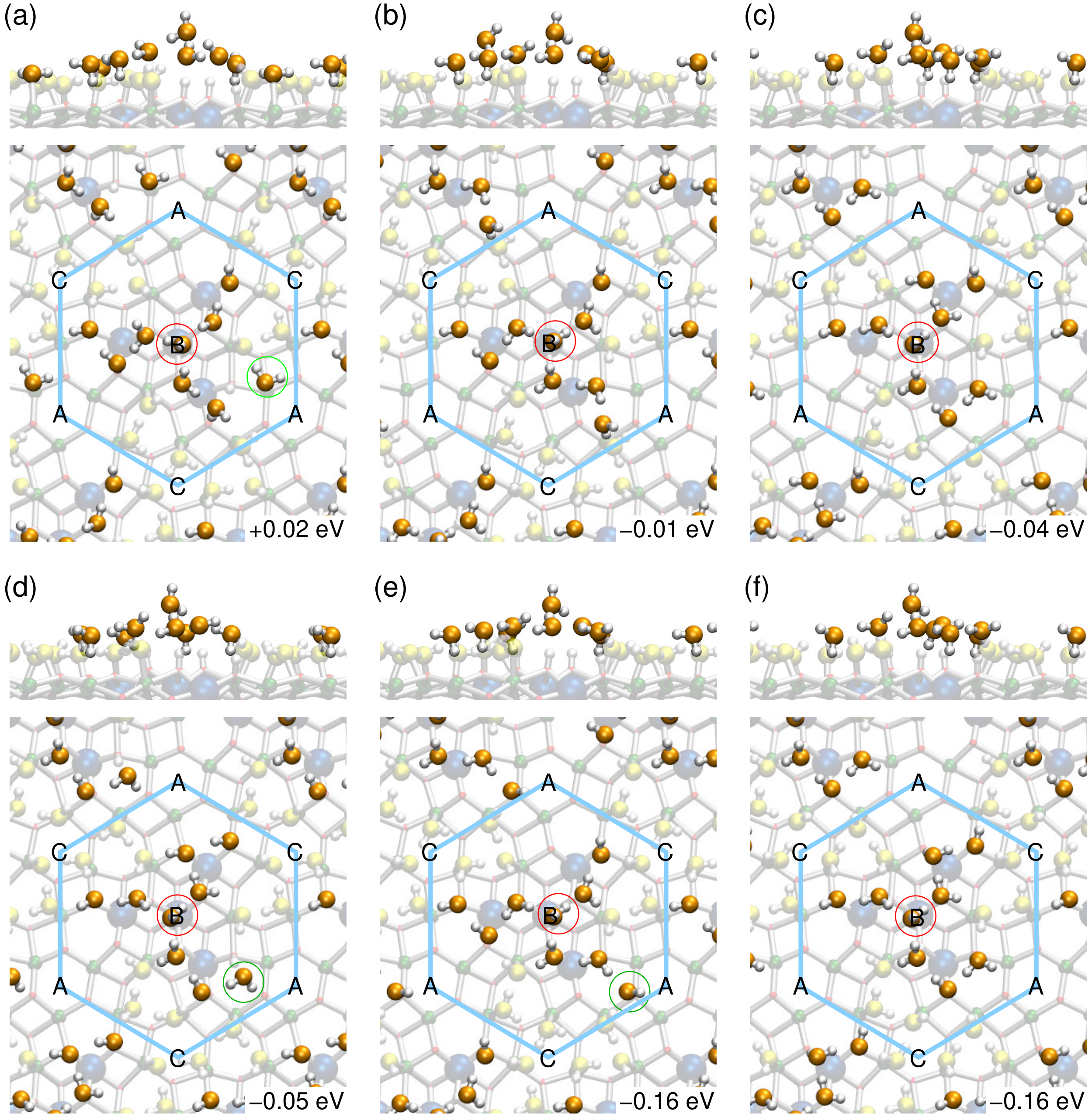

Figure S10. Low-energy structures of 18 water molecules on $In_2O_3(111)$ after manual manipulation of the best structures from the simulated annealing search. The energy differences per unit cell with respect to the global minimum from simulated annealing (Figure S9a) are given in the insets. The same color code as in Figure S9 is used.

9) Structures with 9 Adsorbed Water Molecules (RE Phase)

When the water coverage increases from 6 to 9 molecules in the (1×1) surface unit cell, the adsorbed intact water molecules reorient and the OH groups of the dissociated molecules change position. This rearrangement is the main reason why the structure with 9 water molecules appears as a broad shoulder and not as a separate desorption peak in the TPD spectra. Accordingly, this phase was termed 'reorganization layer' (RE). In the simulated annealing search for low-energy structures of 18 water molecules on the $In_2O_3(111)$ surface it was observed that a fourth water molecule in the first water layer may dissociate. If a fourth dissociated water molecule is already stable at the lower coverage of 9 water molecules, this may further contribute to the 'reorganization' character of this phase. Therefore, we re-investigated the structure of 9 water molecules in the unit cell of the $In_2O_3(111)$ surface[27] to see whether other structures with similar water binding energies may exist.

In our previous DFT investigation of Ref. 27, only water structures preserving the 3-fold symmetry of the surface were considered. Under this constraint, either only 3 or 6 water molecules have to be dissociated. Structures with 6 dissociated molecules were found to be clearly unfavorable.[27] However, close inspection of our proposed symmetric structure of 9 water molecules of Ref. 27 (see Figure S11a) shows that by changing the orientation of one water molecule adsorbed at an In-c site, thereby breaking the 3-fold symmetry, one additional H bond can be formed. DFT calculations show that this additional H bond indeed lowers the overall energy by 0.08 eV per unit cell (see Figure S11c). Also other rearrangements of the H bonds can lead to energy gains of a few hundredths of an eV, see Figure S11b.

In the next step we took the structures with 18 water molecules from the simulated annealing search and removed the 9 water molecules of the second layer, which are not connected to any In surface site. After structure relaxation we found a few configurations with 5 intact and 4 dissociated water molecules, which are within an energy range of 0.12 eV per unit cell of the high-symmetry configuration with only 3 dissociated molecules (see Figure S11d-i). Key for the stabilization of the fourth dissociated water molecule is that the O_wH group receives an H bond from a neighboring intact water molecule. Actually, this H bond triggers the dissociation of the fourth water molecule. A similar dissociation mechanism was observed for water on $ZnO(10\bar{1}0)$, where the interaction within water pairs results in the dissociation of half of the adsorbed water molecules.[9] Therefore, only one more water molecule can dissociate: 5 intact molecules can stabilize 4 O_wH groups by H bonds, but 4 intact molecules are not enough to stabilize 5 O_wH, considering the distribution of the In-c, In-e and In-f sites on the $In_2O_3(111)$ surface.

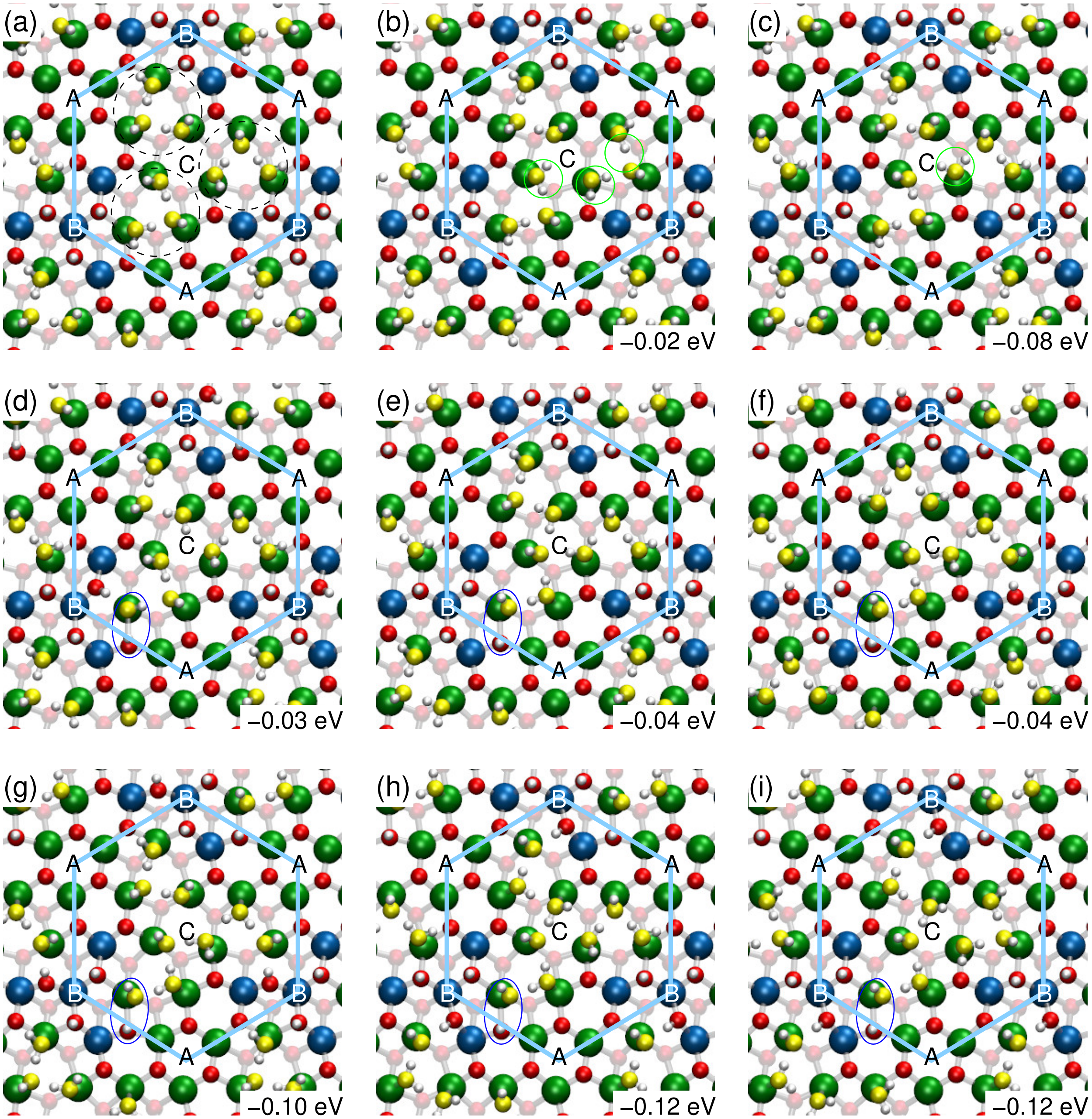

Figure S11. Set of different structures of 9 water molecules on $In_2O_3(111)$ within an energy window of 0.12 eV per unit cell, which give rise to the reorganization phase (RE) in the TPD spectra. The high-symmetry structure of Ref. 27 with 3 dissociated water molecules is shown in (a). The three symmetry-equivalent water trimers are indicated by black dashed circles. In (b,c) an additional H bond is formed by a reorientation of water molecules (marked by green circles), which breaks the 3-fold symmetry of the surface. (d-i) are low energy structures with a fourth dissociated water molecule (blue ellipse). The energy differences per unit cell with respect to the high-symmetry structure (a) are given in the insets.

10) Water Adsorption in Region A

Additional DFT calculations were performed to determine the binding energy of water molecules at In-a sites on $In_2O_3(111)$ surfaces with different water pre-coverage. As already shown in Ref. 27, dissociation of water molecules at In-a and O(α) sites is energetically always less favorable than molecular adsorption. Therefore, in the following only molecular adsorption is considered. Water molecules were placed in various orientations with their O_w atom above an In(5c) of area A. In the geometry optimizations it was observed that the water molecules can adopt 4 different orientations: one of the H atoms points either to a neighboring O(α) or to an O(δ) surface atom and the second H is either clockwise ('CW') or counterclockwise ('CCW') oriented with respect to the first hydrogen (see Figure S12). Structures in which the second H atom points away from the surface were found to be unstable. Water adsorption at In-a sites was studied for surfaces with a pre-coverage of 0, 3, 6 and 9 water molecules in region B and C. The low-energy structures shown in Figure 4 and Figure 5 of the manuscript were taken as initial configurations in the geometry optimizations.

The calculated water binding energies are summarized in Table S2 and the most favorable orientation of the water molecules at each water coverage is shown in Figure S12. Since the directional lone-pair interaction pins the water molecules to their In(5c) sites, distances between water molecules in area A and other water molecules on the pre-covered surfaces are too large for establishing strong hydrogen bonds. The same is true for the interaction between water molecules within the same area A. This is shown by the adsorption of a water trimer on the three available In-a sites. The results in Table S2 show that the binding energy of water molecules at In-a sites is always smaller the lattice energy of bulk ice (0.66 eV), indicating that the growth of bulk ice is favored over adsorption in area A.

orientation	bare	3w	6w	9w
O(α), CW	0.495 (0.423)	0.447	**0.542**	**0.516**
O(α), CCW	0.443 (0.462)	**0.483**	0.540	0.343
O(δ), CW	0.484 (0.479)	0.474	0.430	0.215
O(δ), CCW	**0.564 (0.574)**	0.477	0.540	0.442

Table S2. Binding energy E_b (in eV) for molecular adsorption of single water molecules at In-a sites in the primitive (1×1) unit cell of the $In_2O_3(111)$ surface. The surface is initially either water-free (bare) or pre-covered by 3, 6 or 9 water molecules per unit cell. The four possible orientations of the water molecule are described in the text. Either one In-a site or all three In-a sites in the unit cell (given in parenthesis) are occupied by a water molecule. The lowest-energy structure for each water coverage (bold numbers) is shown in Figure S12.

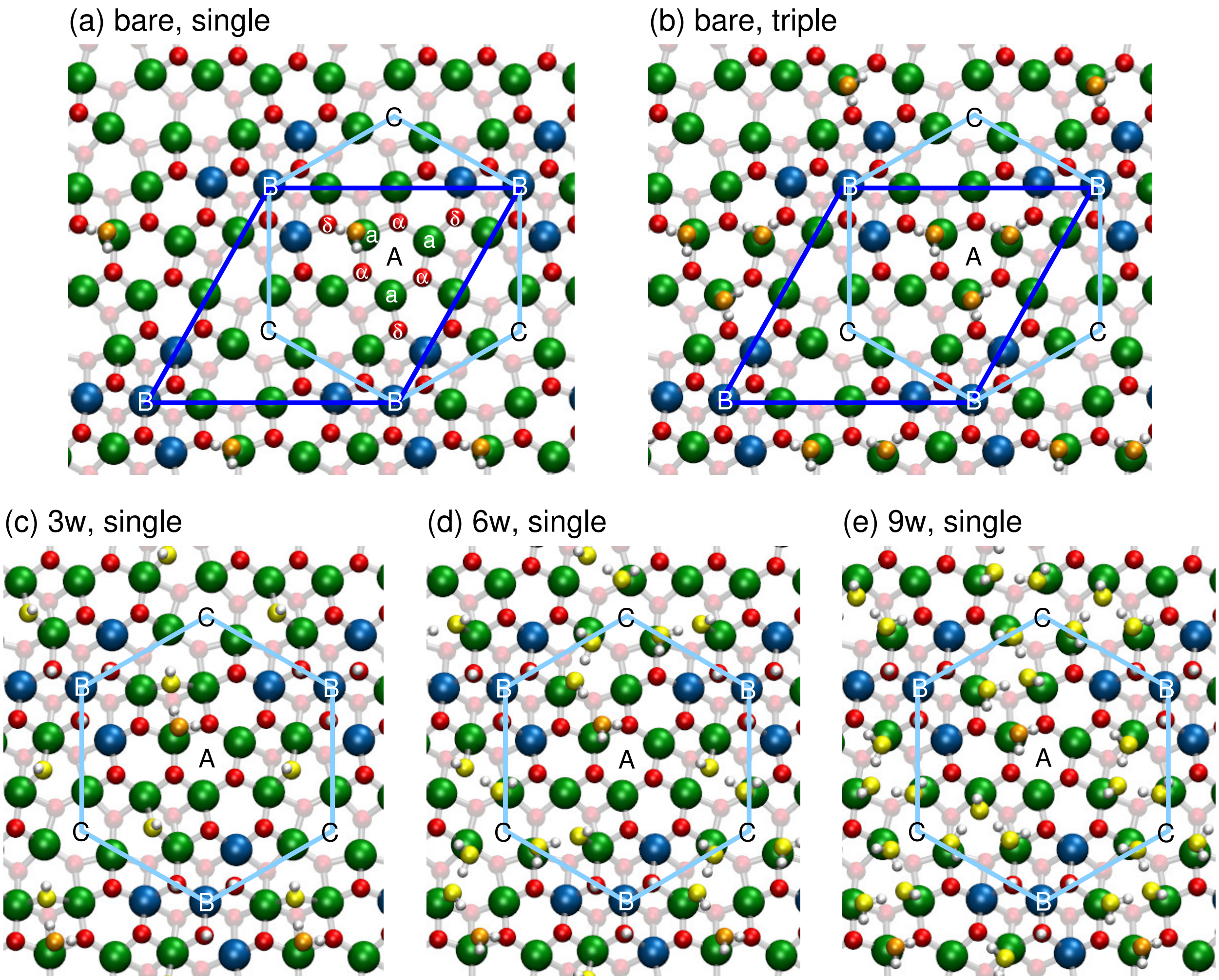

Figure S12. Structure of the most favorable orientation of additional water molecules adsorbed at In-a sites in area A for different water pre-coverages, see bold entries in Table S2. The oxygen atom of the water molecules at In-a sites is shown in orange and those of the water pre-coverage in yellow. Dark and light blue lines show a primitive unit cell and a Wigner-Seitz cell centered at site A, respectively.

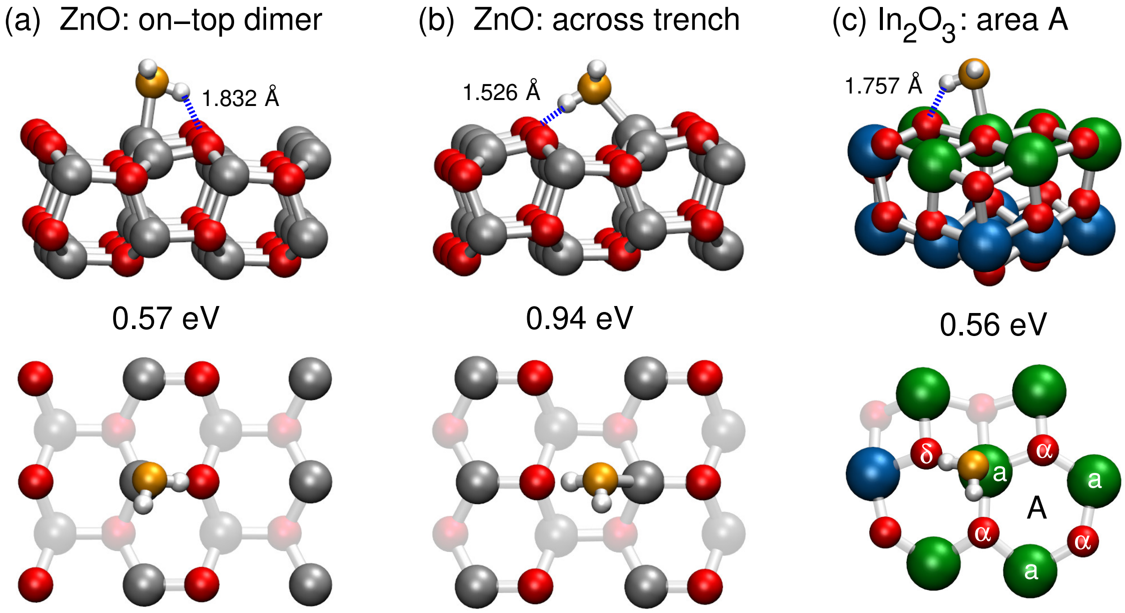

Figure S13. (a,b) Adsorption of a single water molecule on ZnO($10\bar{1}0$). For on-top adsorption in (a), the H bonding is unfavorable, and the binding energy is similar to the one obtained for $In_2O_3(111)$ at In-a in area A shown in (c). For adsorption across the trench in (b), both Lewis acid and base interactions are optimized, which increases significantly the binding energy. Such configurations are not possible in area A of the $In_2O_3(111)$ surface.

11) MD Simulation for a Thick Water Film on $In_2O_3(111)$

For the *ab initio* molecular dynamics simulation of the water/$In_2O_3(111)$ solid/liquid interface, 120 water molecules were added at random positions above the hydroxylated surface. The structure was pre-equilibrated for 1 ns using a force field. The equilibration was continued for 50 ps at 360 K by AIMD using massive Nosé–Hoover thermostats. The final production run of 250 ps at the same temperature was done in the canonical NVT ensemble using Nosé-Hoover chain termostats for the nuclear and electronic degrees of freedom.

The MD trajectory was analyzed for (a) the presence of an adsorbed water molecule on an In(5c) surface sites, (b) the protonation of a surface O(3c), and (c) whether a water molecule has established a H bond to an O(3c) site. Applying a simple distance criterion, the probability of these interactions was determined for the individual types of surface atoms. Plotted in Figure S14 is the fraction of time during the MD simulation at which a water adsorbate, a proton or a H bond are present. For the cutoff distances, the edge of the corresponding peak in the radial distribution functions was taken, see Figure S15. Specifically, the chosen distances are: $d_{\mathrm{InO}} = 2.80$ Å for water adsorption at In(5c), $d_{\mathrm{OH}} = 1.15$ Å for protonation of O(3c), and $d_{\mathrm{HB}} = 2.15$ Å for H bonds to O(3c).

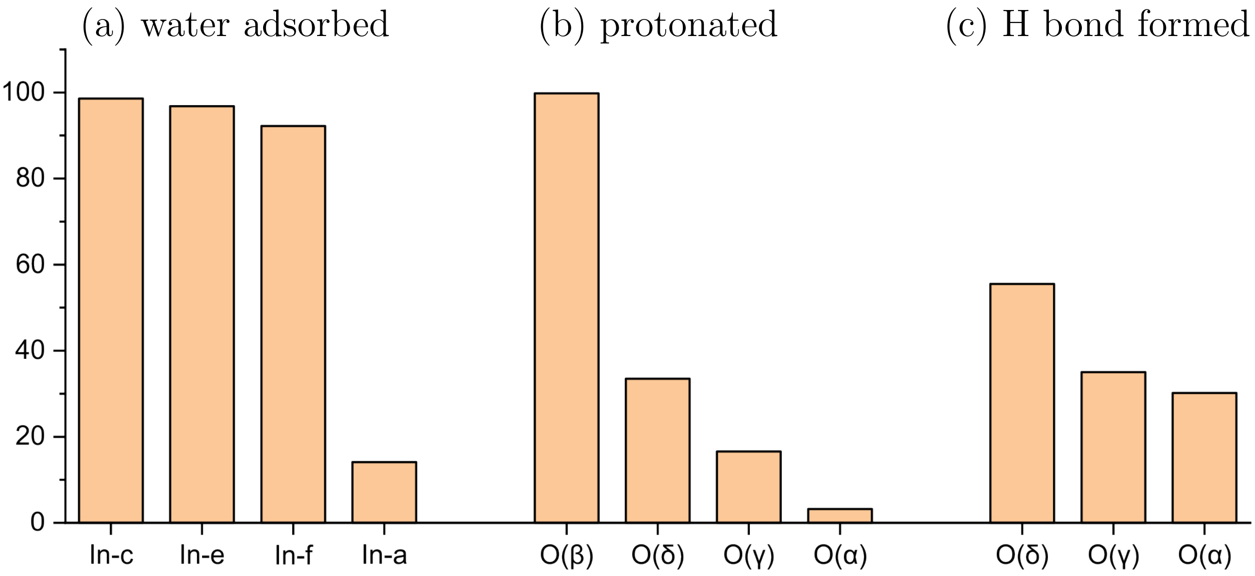

Figure S14. Fraction of time of the 250 ps MD simulation (in %) for which (a) a water molecule is adsorbed at an undercoordinated In(5c) site, (b) an undercoordinated O(3c) of the surface is protonated, or (c) a H bond between a water molecule and an O(3c) site is established. While the In-c, In-e and In-f sites are covered by water molecules (intact or dissociated) basically all the time, the In-a sites remain mostly water-free. All O(β) and one out of the three O(δ) are protonated all along the MD run. Some protonation events by water dissociation take place at O(γ) sites but very rarely at O(α). Hydrogen bonds between water molecules and unprotonated O(3c) surface sites are established for 30–60 % of the time, with the lowest probablity for O(α).

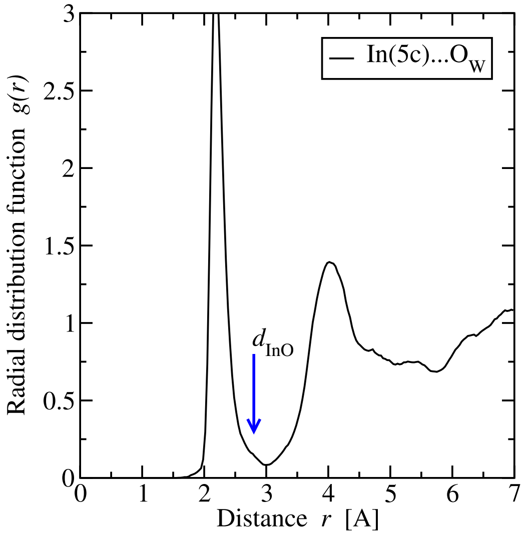

Figure S15. Radial distribution functions $g(r)$ of the surface In(5c) and the water O_W (left) and the surface O_S(3c) and the water H_W (right). The cutoff distances used for the statistical analysis of the MD trajectory are indicated by blue arrows.
